# Accurate Location of Catheter Tip With the Free-to-Total Metanephrine Ratio During Adrenal Vein Sampling

**DOI:** 10.3389/fendo.2022.842968

**Published:** 2022-02-24

**Authors:** Foteini Christou, Edward Pivin, Alban Denys, Karim A. Abid, Tobias Zingg, Maurice Matter, Antoinette Pechère-Bertschi, Marc Maillard, Eric Grouzmann, Gregoire Wuerzner

**Affiliations:** ^1^ Service of Internal Medicine, Lausanne University Hospital and University of Lausanne, Lausanne, Switzerland; ^2^ Service of Nephrology and Hypertension, Lausanne University Hospital and University of Lausanne, Lausanne, Switzerland; ^3^ Department of Radiology, Lausanne University Hospital, Lausanne, Switzerland; ^4^ Laboratoire des Catécholamines et Peptides, Lausanne University Hospital and University of Lausanne, Lausanne, Switzerland; ^5^ Department of Visceral Surgery, Lausanne University Hospital, Lausanne, Switzerland; ^6^ Hypertension Unit, Service of Nephrology and Hypertension, University Hospital Geneva, Geneva, Switzerland

**Keywords:** primary aldosteronism, secondary hypertension, aldosterone, adrenal vein sampling (AVS), metanephrines (plasma), cortisol

## Abstract

**Background:**

The selectivity index (SI) of cortisol is used to document correct catheter placement during adrenal vein sampling (AVS) in patients with primary aldosteronism (PA). We aimed to determine the cutoff values of the SIs based on cortisol, free metanephrine, and the free-to-total metanephrine ratio (FTMR) using an adapted AVS protocol in combination with CT.

**Methods:**

Adults with PA and referred for AVS were recruited in two hypertension centers. The cortisol and free metanephrine-derived SIs were calculated as the concentration of the analyte in adrenal veins divided by the concentration of the analyte in the distal vena cava. The FTMR-derived SI was calculated as the concentration of free metanephrine in the adrenal vein divided by that of total metanephrine in the ipsilateral adrenal vein. The AVS was classified as an unequivocal radiological success (uAVS) if the tip of the catheter was seen in the adrenal vein. The SI cutoffs of each index marker were established using receiver operating characteristic curve analysis.

**Results:**

Out of 125 enrolled patients, 65 patients had an uAVS. The SI cutoffs were 2.6 for cortisol, 10.0 for free metanephrine, 0.31 for the FTMR on the left side, and 2.5, 9.9, and 0.25 on the right side. Compared to free metanephrine and the FTMR, cortisol misclassified AVS as unsuccessful in 36.6% and 39.0% of the cases, respectively.

**Conclusion:**

This study is the first to calculate the SIs of cortisol, free metanephrine, and the FTMR indices for the AVS procedure. It confirms that free metanephrine-based SIs are better than those based on cortisol.

## Introduction

Primary aldosteronism (PA) is one of the most common forms of secondary hypertension (1–3). Identification of patients with this disease is important because they have an increased risk of target organ damage and carry a worse cardiovascular prognosis as compared to patients with essential hypertension with the same level of blood pressure (BP) (4). Two therapeutic strategies may be proposed to patients with PA: blockade of the mineralocorticoid receptor or surgical removal of one adrenal gland if there is lateralized overproduction of aldosterone in one of the adrenal glands. Unilateral adrenalectomy may offer a potential cure for patients with favorable conditions (5, 6).

The standard reference technique to show lateralization of aldosterone secretion is adrenal vein sampling (AVS) (7, 8). However, this procedure is not standardized across reference centers and countries (8). The technique may differ in several ways such as sequential or bilateral simultaneous measurement of hormones, cosyntropin stimulation, or no stimulation. Historically, cortisol with or without cosyntropin stimulation has been used as a marker of correct catheter positioning in the adrenal veins; and, traditionally, adrenal vein cortisol concentration to peripheral vein (antecubital fossa or inferior vena cava below the adrenal veins or iliac vein) plasma cortisol concentration ratio defines the selectivity index (SI). However, several cutoffs have been used to define the SI, which may have limited the use of AVS due to the absence of clear consensus and may have excluded patients from a curative strategy when too stringent cutoffs were applied (9, 10).

The use of free metanephrine (FMN) has been shown to be superior to cortisol in assessing SI, particularly in the setting of non-stimulated sampling of the adrenal veins (11, 12). Sulfotransferase 1A3, the enzyme that sulfate-conjugates FMN, is mainly found in the intestine, but its expression in the adrenal gland has yet to be reported to the best of our knowledge (13). Consequently, FMN, which is released continuously from adrenal chromaffin cells and independently of any stimulation, is then conjugated peripherally (14, 15). We thus hypothesized that the free-to-total metanephrine ratio (FTMR) in the adrenal veins would be a better marker of correct catheter placement than cortisol, like FMN alone, but with the advantage of reducing the number of sampling sites during AVS. Indeed, the closer the vein sampling is to the adrenal gland, the higher the FTMR will be.

The first objective of the study was to determine SI cutoffs for the FTMR, FMN, and cortisol using a contrast-enhanced multi-detector CT. An adapted AVS protocol allowed us to perform receiver operating characteristic (ROC) analysis. The second objective was to compare the performance of cortisol, FMN, and the FTMR for the successful assessment of adrenal vein catheterization.

## Methods

This was a prospective study conducted from 2013 to 2018 with patients from two reference centers for hypertension: the Geneva University Hospital and the Lausanne University Hospital. Eligible participants were men and women older than 18 years with a diagnosis of PA based on two increased aldosterone/plasma renin activity ratios or one increased ratio associated with a concomitant increased urinary 24-h aldosterone excretion and referred for an AVS at the Lausanne University Hospital. No confirmatory suppression test was carried out, as lateralization of aldosterone secretion is not excluded by the positive saline infusion suppression test (16). A single experienced interventional radiologist performed all procedures in a hybrid interventional operating room equipped with a 128-slice CT Philips Ingenuity (Philips Systems, Cleveland, OH, USA) and C-Arm Philips Veradius Unity (Philips Systems, Cleveland, OH, USA). Each patient was lying down on the table of the CT scan. After rigorous asepsis and surgical field placement, a common right femoral 6F access was introduced using the Seldinger technique, under local anesthesia and with US guidance. Under fluoroscopy, the radiologist catheterized each adrenal vein and the proximal and the distal vena cava and obtained blood samples. The procedure was performed sequentially without cosyntropin stimulation. The order of catheterization and blood sampling was the proximal vena cava (suprarenal), the distal vena cava (infrarenal), the left adrenal vein, and finally the right adrenal vein. The position of the tip of the catheter was confirmed by gently injecting contrast media to show retrograde adrenal parenchymography for both sides. For the right side, a small helical CT acquisition was also conducted to prove the placement of the catheter in the right adrenal vein. When the operator had difficulties locating the adrenal veins, additional CT acquisitions were performed (**Supplementary Figure 1**). The procedure was conducted in an outpatient setting. Antihypertensive drugs other than the alpha-blocker doxazosin or calcium channel blockers were stopped 2 weeks prior to AVS, except for spironolactone, which was stopped 6 weeks before the procedure.

The study was approved by the local ethics committee (Commission cantonale d’éthique de la recherche sur l’être humain, www.cer-vd.ch). Each participant provided written informed consent. The procedures followed in this study were in accordance with institutional guidelines.

Plasma renin activity (PRA) and plasma aldosterone were measured with commercial radioimmunometric assays (RENCTK (Angiotensin I) RIA, DiaSorin, Saluggia, Italy, and ALDO RIACTR Kit, Cisbio Bioassays, Codolet, France, respectively). Serum cortisol levels were measured with fluorescence polarization immunoassay (FPIA) on an AxSYM instrument (Abbott Diagnostics, Lake Forest, IL, USA). Plasma free and total metanephrines were measured using ultrahigh-performance liquid chromatography–tandem mass spectrometry (17, 18).

The cortisol-derived SI was calculated as the concentration of cortisol in adrenal vein samples divided by that in distal vena cava samples. The FMN-derived SI was calculated as the concentration of FMN in adrenal vein samples divided by that in distal vena cava samples. The FTMR-derived SI was calculated as the concentration of FMN in adrenal vein samples over total metanephrine in the ipsilateral adrenal vein. The SI cutoffs of each index marker were established using ROC curve analysis. Placement of the tip of the catheter in the proximal vena cava confirmed by angiography was defined as a negative test, and placement of the tip of the catheter in the adrenal vein confirmed by angiography coupled with CT was defined as a positive test for the reference test. We used the FTMR in the proximal vena cava and the proximal vena cava to distal cava cortisol or FMN ratio as the corresponding negative index test. The AVS was classified as an unequivocal radiological success (uAVS) if the radiologist had the visual certainty of correct adrenal vein placement using angiography coupled with CT. If the success of the adrenal vein catheterization was doubtful, the procedure was classified as equivocal radiological success (eAVS).

### Adrenal Gland Metanephrine Content

Free and total metanephrines (i.e., free + sulfated metanephrine) were extracted from tissues of four human adrenal glands obtained from kidney transplant organ donors after disruption in perchloric acid 0.1 M and were sonicated using a Branson Sonifier 450 (Branson, Danbury, CT, USA) at full power for 30 s. Sulfated metanephrines were hydrolyzed with sulfatase from *Aerobacter aerogenes* (S1629) purchased from Sigma-Aldrich (St. Louis, MO, USA) as previously reported (19). Values are expressed in nanomoles of metanephrine per gram of tissue.

### Statistics

Data are presented as means ± SD or medians with interquartile range (IQR) if variables were non-normally distributed. A t-test was used to compare normally distributed variables. A rank-sum test was used to compare data not normally distributed. A chi-square test was used to compare categorical variables. ROC curve analysis was used to establish the most appropriate SI for each marker in the subset of patients with uAVS. The point chosen to establish the SIs was the point providing the maximum sensitivity for a specificity of 100%. Agreement between index markers was tested with a kappa test in the subset of patients with eAVS. Statistical analysis was performed using Stata 15.0 (Stata Corp), and the nominal level of statistical significance was set at a *p-*value of <0.05.

## Results

**Figure 1:**
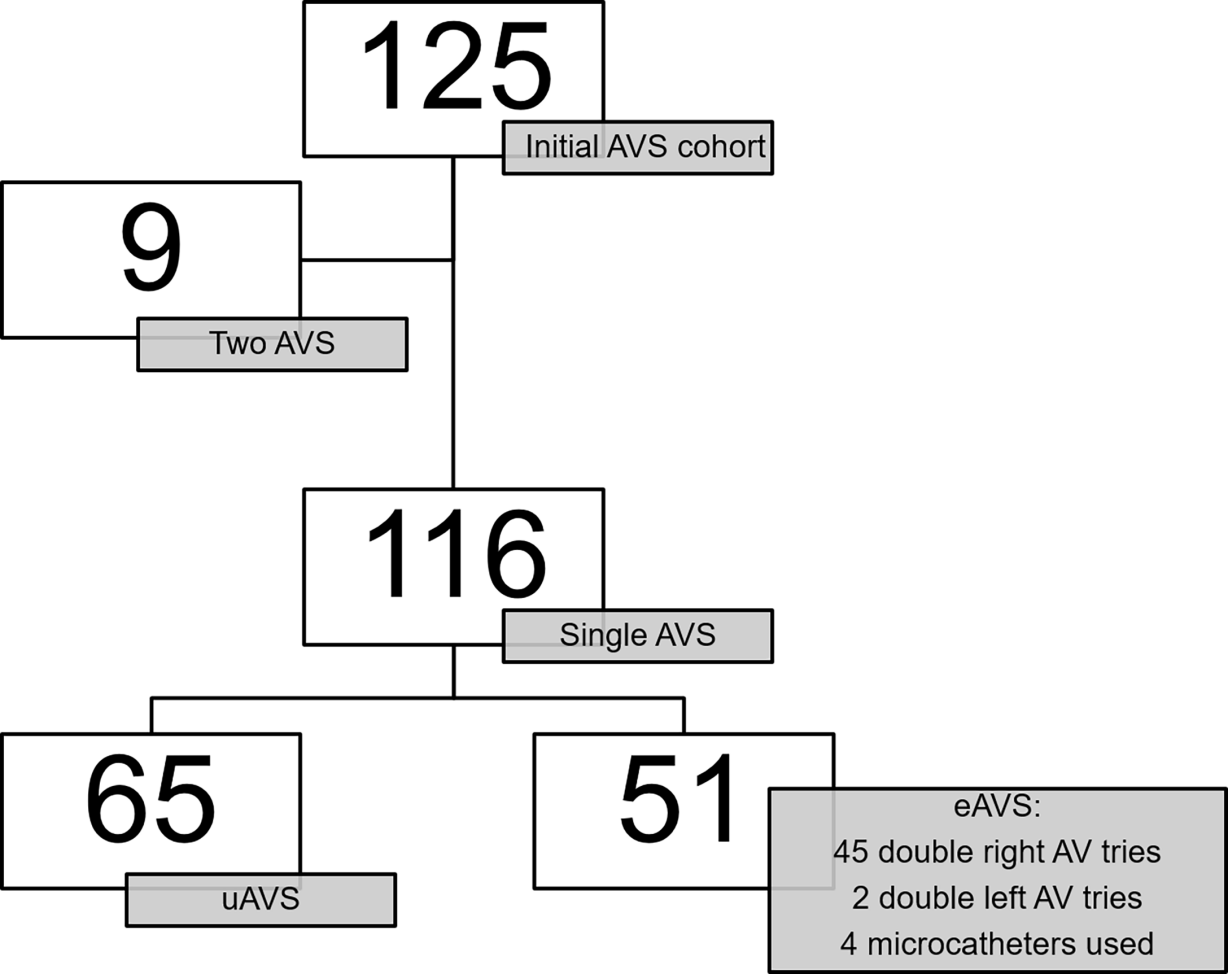
Flowchart. uAVS, radiological unequivocal adrenal vein sampling; eAVS, radiological equivocal adrenal vein sampling; AV, adrenal vein.

Between 2014 and 2018, 125 patients with PA were referred for an AVS and signed their informed consent (**Figure 1**, flowchart). The percentage of patients with normal CT was 45.6%, while 30.4% had a left nodule, 16.0% had a right nodule, 3.2% had left hyperplasia, 1.6% had bilateral nodules, and 2.4% had bilateral hyperplasia. Nine patients were excluded from the analysis because they had a previous unsuccessful (prior to 2013) AVS, suggesting a potential difficult procedure. Of the remaining 116 patients, 65 had radiological unequivocal bilateral successful AVS and were used to determine cutoff values of markers of selectivity, while 51 had radiologically equivocal bilateral successful AVS and were used together with 9 patients, who were referred for a second try, in the secondary analysis to compare the performance of the different analytes. Of these 51 participants, the uncertainty of correct placement of the tip of the catheter in the right adrenal vein by the radiologist was the most common reason for eAVS.

Patients’ characteristics are shown in **Table 1**. More than half of the participants were men, and the majority were of Caucasian ethnicity. Despite the use of oral potassium in patients with hypokalemia diagnosed before AVS, mean serum potassium was still below the local laboratory reference range (3.5–4.5 mmol/L).

**Table 1:** Baseline patients’ characteristics.

	Total (n = 116)	uAVS (n = 65)	eAVS (n = 51)	*p*-Value
Number	116	65	51	
Sex (women, %)	50 (43)	28 (43)	22 (43)	0.995
Ethnicity (Caucasian, %)	101 (87.1)	55 (84.6)	46 (90.2)	0.791
Age (years)	49.2 ± 10.2	50.5 ± 10.2	47.6 ± 10.1	0.129
BMI (kg/m^2^)	30.0 ± 6.9	30.0 ± 6.6	30.1 ± 7.5	0.949
Office SBP (mmHg)	151.9 ± 17.7	153.8 ± 18.8	149.4 ± 15.8	0.185
Office DBP (mmHg)	92.8 ± 12.7	93.7 ± 12.4	91.7 ± 13.1	0.4
Office heart rate (bpm)	73.5 ± 12.1	74.7 ± 12.3	71.7 ± 11.7	0.229
eGFR (ml/min/1.73 m^2^)	87.9 ± 22.5	87.4 ± 22.5	88.5 ± 23.4	0.8
Plasma K^+^ (mmol/L)	3.35 ± 0.45	3.31 ± 0.41	3.40 ± 0.49	0.266
PRA (ng/ml/h)	0.1 (0.10–0.26)	0.1 (0.10–0.19)	0.1 (0.10–0.4)	0.039
Aldosterone (pg/ml)	145 (94–225)	145 (94–194)	141 (93–241)	0.824

Data are n (%), median (IQR), and mean (SD), unless otherwise specified.

uAVS, unequivocal radiologic successful adrenal vein sampling; eAVS, equivocal radiologic successful adrenal vein sampling; SBP, systolic blood pressure; DBP, diastolic blood pressures; eGFR, estimated glomerular filtration rate; PRA, plasma renin activity.

### Hormonal Concentration at Specific Sampling Site (Distal Vena Cava, Proximal Vena Cava, Left Adrenal Vein, Right Adrenal Vein, and Adrenal Gland)

**Figure 2:**
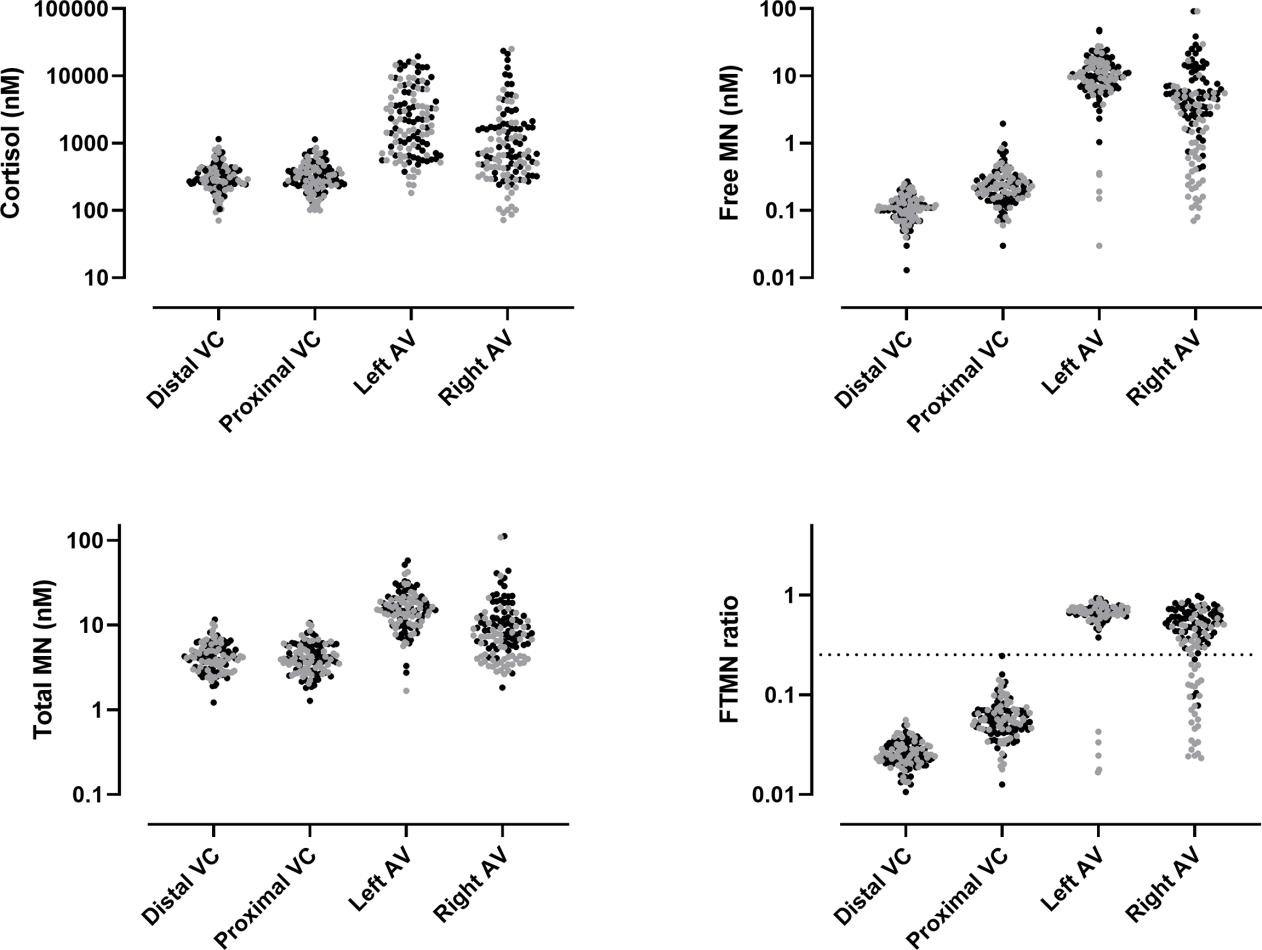
Hormonal concentrations and free/total metanephrine ratio (FTMR) at specific sites with calculated selectivity index for FTMR (dashed line). VC, vena cava; AV, adrenal vein; MN, metanephrine; AVS, adrenal vein sampling. Black dot: unequivocal AVS. Gray dots: equivocal AVS.

Hormonal concentrations at specific sampling sites are shown in **Table 2** for patients with uAVS and eAVS. In patients with uAVS, aldosterone, FMN, and the FTMR were all higher in the proximal vena cava than in the distal vena cava. Conversely, total metanephrine was higher in the distal vena cava. The median ratios of the right adrenal vein to the distal vena cava of cortisol, FMN, and total metanephrine were respectively 3.0 (1.6–7.2), 54.5 (27.4–107.5), and 2.3 (1.7–4.0) (**Supplementary Figure 2**). The ratios from the left adrenal vein were 6.3 (3.1–149), 100.3 (72.8–130.8), and 3.6 (2.8–4.5). The ratios from the left side were significantly higher, except for total metanephrine. In the adrenal gland, 96.8 (median) of the total metanephrine consisted of FMN (**Table 2**). Hormonal concentrations and ratios from the whole cohort (equivocal and unequivocal AVS) are shown in **Figure 2**.

**Table 2:** Adrenal, adrenal veins, and proximal and distal vena cava plasma concentrations.

	Group	Distal VC	Proximal VC	Left AV	Right AV	Adrenal gland (N = 4)
Aldosterone (pg/ml)	uAVS	145 (94; 194)	179 (116; 340)*	1,263 (711; 4,687)	834 (462; 1,700)	.
	eAVS	140 (93; 251)	166 (95; 351)	1,138 (407; 6,227)	294 (84; 1,205)	
Cortisol (nmol/L)	uAVS	307 (252; 416)	339 (256; 433)*	2,235 (887; 6,408)	1,184 (515; 2,040)	.
	eAVS	280 (211; 394)	290 (201; 415)	1,671 (689; 4,011)	507 (295; 1,169)	
Free metanephrine (nM)	uAVS	0.11 (0.08; 0.13)	0.22 (0.15; 0.29)*	10.0 (6.6; 15.1)	5.54 (3.1; 12.2)	17.8 (12.5; 20.7)
	eAVS	0.11 (0.09; 0.14)	0.23 (0.18; 0.32)	9.55 (6.94; 13.3)	1.56 (0.3; 4.65)	
Total metanephrine (nM)	uAVS	4.33 (3.06; 5.56)	4.18 (2.89; 5.82)*	15.0 (9.9; 21.5)	10.2 (6.5; 18.3)	18.1 (12.5; 21.0)
	eAVS	4.06 (3.1; 5.24)	3.90 (3.1; 5.06	14.9 (10.8; 18.8)	6.3 (3.8; 9.13)	
FTMR	uAVS	0.025 (0.021; 031)	0.054 (0.043; 0.069)*	0.69 (0.634; 0.729)	0.57 (0.430; 0.694)	0.968 (0.903; 1.02)
	eAVS	0.025 (0.022; 0.031)	0.056 (0.045; 0.072)	0.688 (0.626; 0.749)	0.3 (0.086; 0.495)	

Data are medians with interquartile ranges.

VC, vena cava; AV, adrenal vein; uAVS, unequivocal adrenal vein sampling; eAVS, equivocal adrenal vein sampling.

*p < 0.05 distal VC vs. proximal VC in patients with uAVS.

### Selectivity Indices for Cortisol, Free Metanephrine, and the Free/Total Metanephrine Ratio

Using ROC curves analysis, with CT-confirmed correct catheter tip placement used as the reference test (positive if the catheter is in the adrenal vein and negative if the catheter is in the proximal vena cava), the area under the curve was 1.0 (95% CI 1.000–1.000) for FMN, 1.0 (95% CI 1.000–1.000) for the FTMR, and 0.913 (95% CI 0.981–1.000) for cortisol on the left side. The area under the curve was 0.998 (95% CI 0.990–1.000) for FMN, 0.995 (95% CI 0.988–1.000) for the FTMR, and 0.882 (95% CI 0.813–953) on the right side. ROC curves are shown in **Supplementary Figure 3**. The SI cutoff values of each marker are shown in **Table 3**.

**Table 3:** Range and cutoff values for selectivity indices for cortisol, free metanephrine, and the free-to-total metanephrine ratio.

	Range	Cutoff value	Sensitivity%	95% CI	Specificity%	95% CI
Cortisol left	0.635–83.7	>2.63	79.0	66.8% to 88.3%	100	94.5% to 100.0%
Cortisol right	0.502–241	>2.50	57.8	44.8% to 70.1%	100	94.5% to 100.0%
Free MN left	0.75–579	>10.00	100.0	94.5% to 100.0%	100	94.5% to 100.0%
Free MN right	0.727–1,014	>9.92	95.4	87.1% to 99.0%	100	94.5% to 100.0%
Free/total MN ratio left	0.167–0.937	>0.31	100.0	94.5% to 100.0%	100	94.5% to 100.0%
Free/total MN ratio right	0.233–0.984	>0.25	93.9	85.0% to 98.30%	100	94.5% to 100.0%

The cortisol-derived SI was calculated as the concentration of cortisol in adrenal vein samples divided by that in distal vena cava samples. The free metanephrine-derived SI was calculated as the concentration of free metanephrine in adrenal vein samples divided by that in distal vena cava samples. The FTMR-derived SI was calculated as the concentration of free metanephrine in adrenal vein samples divided by that of total metanephrine in ipsilateral adrenal vein samples. The SI cutoffs of each index marker were established using receiver operating characteristic curve analysis.

MN, metanephrine; AV, adrenal vein; SI, selectivity index.

### Concordance and Agreement Between Markers of Selectivity in Participants With Equivocal Radiological Success

Bilaterally successful and unsuccessful AVS based on the newly defined cutoffs for each selectivity marker and applied to the group with eAVS (51 patients) and those with double AVS (9 patients) is shown in **Table 4**. The use of FMN and the FTMR as selectivity markers enabled the reclassification of bilateral AVS wrongly labeled as unsuccessful based on cortisol SI in 36.6% and 39.0%, respectively. Bilateral disagreement between the FMN and the FTMR was found in two cases in which FMN labeled AVS as unsuccessful and where the FTMR labeled AVS as successful. The levels of agreement between cortisol and FMN were 68.3% with a kappa of 0.367 ± 0.120. The level of agreement between cortisol and the FTMR was 68.3% with a kappa of 0.382 ± 0.117. The level of agreement of FMN with the FTMR was 96.7% with a kappa of 0.933 ± 0.129.

**Table 4:** Agreement between the markers of selectivity in the right, left, and both AVS.

		Free MN		FTMR		Side
		Failure	Success	Failure	Success	
Cortisol	Failure	26	15	25	16	Both
	Success	4	15	3	16	
FTMR	Failure	28	0			
	Success	2	30			
Cortisol	Failure	22	17	23	16	Right
	Success	3	18	4	17	
FTMR	Failure	25	0			
	Success	2	33			
Cortisol	Failure	4	12	4	12	Left
	Success	1	43	1	43	
FTMR	Failure	5	0			
	Success	0	55			

FTMR, free-to-total metanephrine ratio; MN, metanephrine; AVS, adrenal vein sampling.

## Discussion

This study is the first to report and compare the SIs of cortisol, FMN, and the FTMR using contrast-enhanced multi-detector CT as a reference test for positivity. Using a modified AVS protocol, which included an additional blood sampling in the proximal part of the vena cava, we were also able to collect data reflecting a true negative test. The study confirms that FMN is a better analyte than cortisol to confirm the successful placement of the catheter’s tip during AVS. In addition, it shows that the FTMR performs as well as FMN and does not necessitate an additional peripheral sample (infrarenal inferior vena cava) for SI determination.

AVS is the reference test to determine lateralization of aldosterone secretion, although some have questioned its superiority in terms of outcomes after surgery when compared to CT (20). As such, the successful placement of the catheter tip in the draining adrenal vein is of paramount importance, and it is usually assessed using cortisol SI. However, no consensus exists regarding the SI of cortisol, and experts advise choosing an SI superior to two or three at the expense of excluding the right positioning of the catheter (21). We show from ROC curve analysis that the SI for cortisol is >2.6 for the left adrenal vein and >2.5 for the right adrenal vein. Interestingly, this cutoff value is very close to the cutoff value of 2.7, which was determined in a multicentric study focusing on the reproducibility of subtype diagnosis in patients with PA, who had two AVS, using different cutoffs (22). The SI of FMN of 9.9 for the right adrenal vein and 10.0 for the left adrenal vein is only marginally lower than the SI proposed by Dekkers et al., who calculated an SI of 12 (11). Their methodology differed from ours since they used a cosyntropin-stimulated adrenal vein/peripheral vein ratio of FMN to assess the selectivity AVS, with a cortisol-derived SI of 3.0 as a reference index. The definite proof of the superiority of FMN or FTMR-derived SI would be to study the biochemical and clinical outcomes in patients with discordant selectivity results with cortisol and these two other markers.

**Figure 3:**
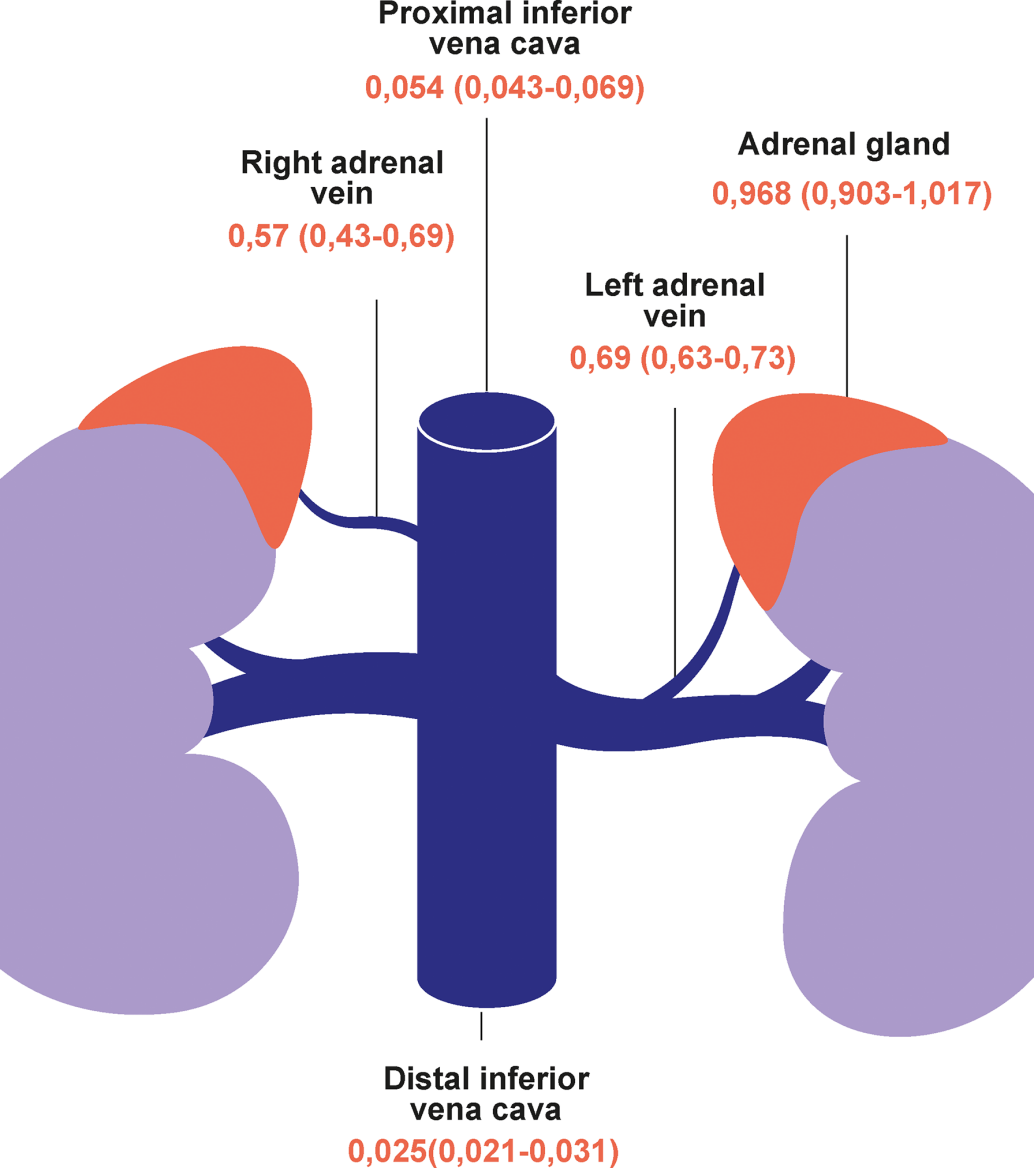
The free-to-total metanephrine ratio from the adrenal glands to the vena cava. Data are median and interquartile ranges.

The FTMR is an interesting marker of selectivity since we show that almost all the metanephrines (>96%) in the adrenal gland are composed of FMN. This ratio progressively decreases along the venous drainage with secondary dilution by peripheral venous circulation and supply of sulfated metanephrine (**Figure 3**) (23). We show for the first time that an FTMR of >0.25 in the right adrenal vein and a ratio >0.31 in the left adrenal vein are sensitive and specific indicators of selectivity.

Historically, cortisol with or without cosyntropin stimulation has been used as the reference analyte for the SI. Cortisol levels are however dependent on stress during the procedure in unstimulated conditions. Secondly, co-secretion of cortisol in PA may influence the SI and the lateralization (21). Subclinical hypercortisolism is not rare, and a prevalence rate of 28% has been reported in the Asian population especially (24–26). Co-secretion may induce negative feedback on the contralateral adrenal gland and therefore generate a false negative SI. It may also decrease the lateralization index on the affected side and result in a false-negative test of lateralization (21). In our study, we confirm that FMN has a better sensitivity than cortisol in determining correct catheter tip location in the adrenal veins in non-stimulated conditions and show that the FTMR performs similarly to FMN with a very good agreement between the two methods. This improved sensitivity, compared to cortisol, allowed the reclassification of bilateral success in 36% (FMN) and 39% (FTMR) of cases classified as bilaterally unsuccessful with cortisol.

The FTMR as the SI needs only two sites of sampling (both adrenals veins) instead of three (both adrenal veins and a peripheral site). It may reduce the duration of the procedure. However, additional laboratory work needs to be performed, and expenses increase as both free and total metanephrines have to be measured. Steroids other than cortisol, such as 17-alpha-hydroxyprogesterone and particularly androstenedione, have been proposed as SI markers, as they have a higher step-up between the adrenal vein and the inferior vena cava than cortisol (12, 27). Indeed, Ceolotto et al. have recently compared the SI of cortisol, FMN, and androstenedione using a bilateral simultaneous unstimulated technique. They have also shown the superiority of FMN and androstenedione over cortisol, allowing the use of AVS data despite an AVS incorrectly labeled as unsuccessful with cortisol. In their comparison, they have, however, used a single arbitrarily but recognized SI cutoff of ≥2 for all analytes. Local expertise in steroids or metanephrine assays may guide the choice of the selectivity marker.

Of note, the ratios of analytes between the adrenal veins and the peripheral veins (distal vena cava) were higher on the left side (**Table 2**), which probably reflects easier and more distal cannulation of the left side. This phenomenon has been observed in some but not all studies (11, 12, 27). In addition, total metanephrine was higher in the distal vena cava than in the proximal vena. This inversion of proportion may be explained by the fact that total metanephrine is more diluted in the suprarenal vena cava.

The monocentric location of the AVS procedure may limit the external validation of the study. Patients were however included in two different tertiary referral hypertension centers, and their clinical characteristics were similar to other studies including patients referred for AVS (11, 27). In addition, the results concerning the SI of FMN are in good agreement with the study of Dekkers et al. (11). A possible limitation of the study was that a confirmatory test such as a saline load was not performed systematically in all patients as recommended by some guidelines (7). The guidelines, however, recognize that the evidence for a confirmatory test is low (7). In addition, this confirmatory test would not have influenced the results since the objective of the study was to determine the cutoff values of cortisol, FMN, and the FTMR. Another limitation of this study is that cosyntropin was not used in this study, limiting the comparison to unstimulated AVS only. The use of cosyntropin during the AVS procedure adds, however, a level of complexity and time to a procedure already known to be challenging (8).

In summary, the FTMR is an SI with high sensitivity and specificity with very good agreement compared to FMN. Both have a better sensitivity than cortisol in unstimulated AVS. In addition, precise cutoffs for all markers were established using a dedicated procedure, compared to the arbitrary cutoffs that have been used so far for cortisol.

## Data Availability Statement

The raw data supporting the conclusions of this article will be made available by the authors upon request.

## Ethics Statement

The studies involving human participants were reviewed and approved by CER-VD. The patients/participants provided their written informed consent to participate in this study.

## Author Contributions

FC captured the data and wrote and reviewed the manuscript (co-first author). EP made the statistical analysis and reviewed the manuscript (co-first author). AD performed the AVS and reviewed the manuscript. KA analyzed the adrenal content. TZ and MauM sampled the adrenal gland from deceased kidney donor and reviewed the manuscript. AP-B selected the patients and reviewed the manuscript. MarM performed the aldosterone and PRA analyses and reviewed the manuscript. EG performed the metanephrine analysis and reviewed the manuscript. GW designed the protocol, selected the patients, supervised the statistical analysis, and wrote the manuscript. All authors listed have made a substantial, direct, and intellectual contribution to the work and approved it for publication.

## Funding

The Swiss National Science Foundation has supported this research with grant nos. PZ00P3_121655 and PZ00P3_137262.

## Conflict of Interest

The authors declare that the research was conducted in the absence of any commercial or financial relationships that could be construed as a potential conflict of interest.

## Publisher’s Note

All claims expressed in this article are solely those of the authors and do not necessarily represent those of their affiliated organizations, or those of the publisher, the editors and the reviewers. Any product that may be evaluated in this article, or claim that may be made by its manufacturer, is not guaranteed or endorsed by the publisher.

## Supplementary Material

Supplementary Figure 1
**(A)** Axial computed tomography angiography of the right adrenal gland. **(B)** Coronal computed tomography angiography of the right adrenal gland.

Supplementary Figure 2 Comparison of selectivity indices (SI) in **(A)** The left adrenal vein. **(B)** The right adrenal vein. **(C)** Both adrenal veins. Total metanephrine (TMN); free metanephrine (FMN). * indicates p<0.05.

Supplementary Figure 3 Receiver operation characteristic curves analysis exploring the diagnostic performance of cortisol, free metanephrine and the free to total metanephrine ratio. AV, adrenal vein; VC, vena cava; MN, metanephrine.

## Abbreviations

AVS, adrenal vein sampling; eAVS, equivocal radiological success of adrenal vein catheterization; uAVS, unequivocal radiological success of adrenal vein catheterization; BP, blood pressure; FMN, free metanephrine; FTMR, free-to-total metanephrine ratio; PA, primary aldosteronism; ROC, receiver operating characteristic; SI, selectivity index.
