# Supplementary figures and images for: Accurate Location of Catheter Tip With the Free-to-Total Metanephrine Ratio During Adrenal Vein Sampling
Source: Front Endocrinol (Lausanne). 2022 Feb 24;13:842968. doi: 10.3389/fendo.2022.842968 (PMC8907625; doi:10.3389/fendo.2022.842968)

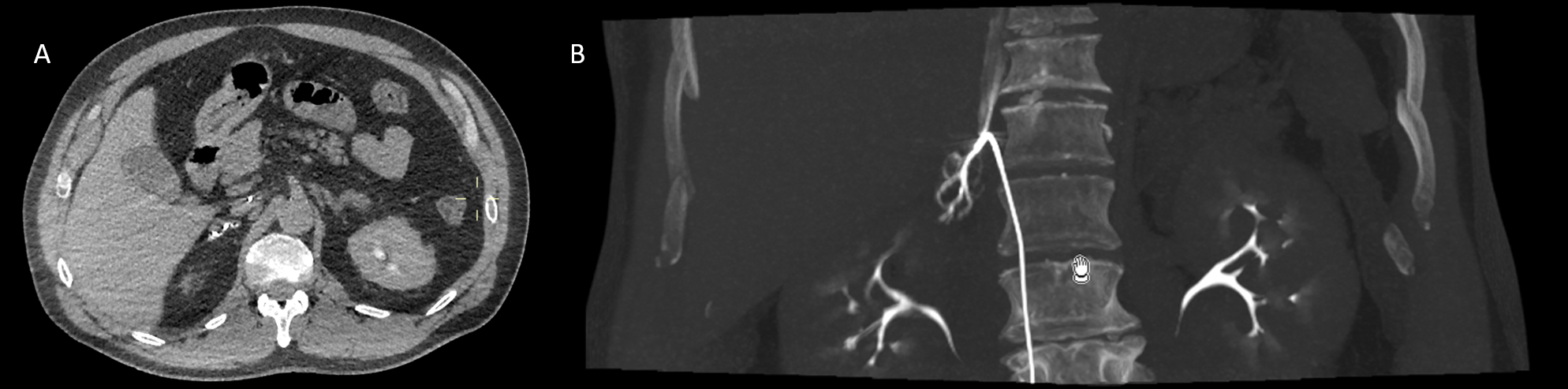

Supplement: Supplementary Figure 1 — (A) Axial computed tomography angiography of the right adrenal gland. (B) Coronal computed tomography angiography of the right adrenal gland. [file Image_1.tif]

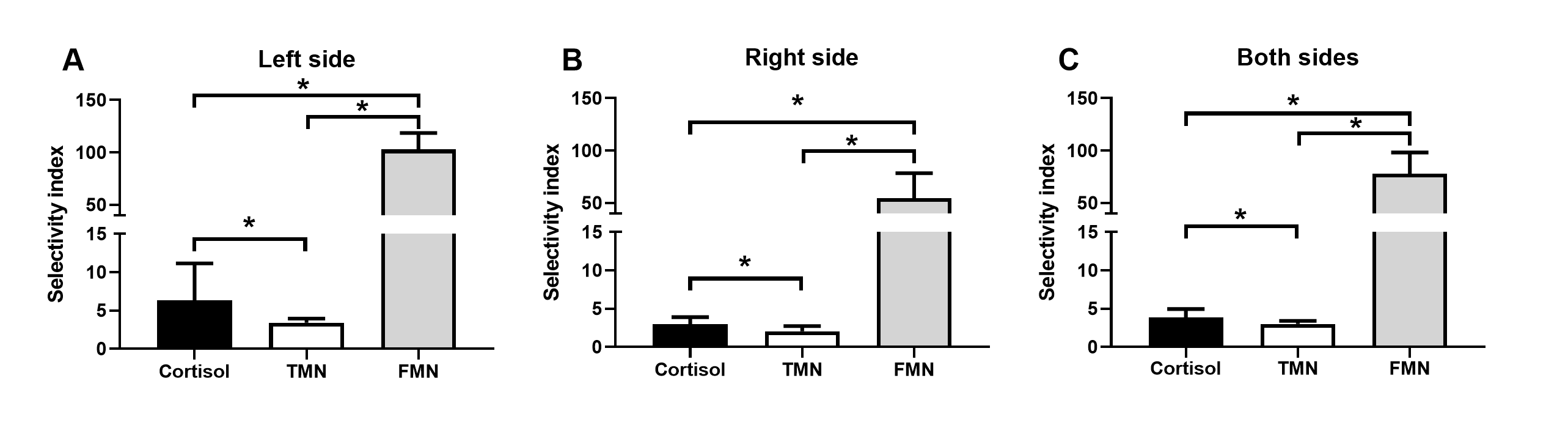

Supplement: Supplementary Figure 2 — Comparison of selectivity indices (SI) in (A) The left adrenal vein. (B) The right adrenal vein. (C) Both adrenal veins. Total metanephrine (TMN); free metanephrine (FMN). * indicates p<0.05. [file Image_2.tif]

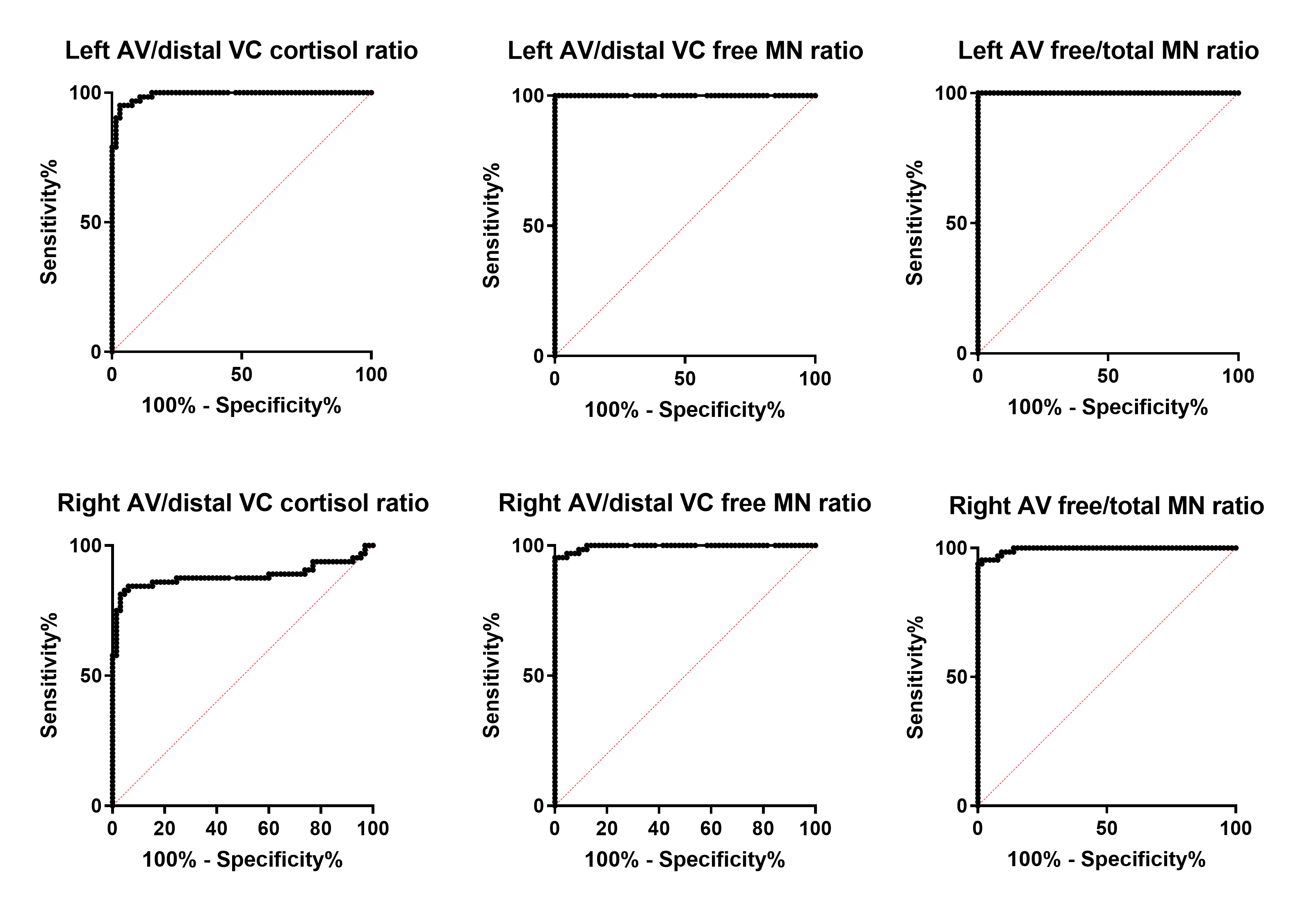

Supplement: Supplementary Figure 3 — Receiver operation characteristic curves analysis exploring the diagnostic performance of cortisol, free metanephrine and the free to total metanephrine ratio. AV, adrenal vein; VC, vena cava; MN, metanephrine. [file Image_3.tif]
